# Noninvasive Follicular Thyroid Neoplasm With Papillary-Like Nuclear Features: What a Surgeon Should Know

**DOI:** 10.7759/cureus.33649

**Published:** 2023-01-11

**Authors:** Jabir Alharbi, Thamer Alraddadi, Haneen Sebeih, Mohammad A Alessa, Haddad H Alkaf, Ahmed Bahaj, Sherif K Abdelmonim

**Affiliations:** 1 Head, Neck and Skull Base Center, King Abdullah Medical City, Makkah, SAU; 2 Otorhinolaryngology - Head & Neck, Prince Mohammad Bin Abdulaziz Hospital Ministry of National Guard, Madinah, SAU; 3 Otorhinolaryngology - Head & Neck Surgery, King Salman Medical City, Madinah, SAU; 4 Otolaryngology - Head and Neck Surgery, Ain Shams University, Cairo, EGY

**Keywords:** thyroidectomy, papillary thyroid carcinoma, noninvasive, lobectomy, fine needle aspiration cytology, follicular thyroid neoplasm

## Abstract

The inclusion of the less aggressive follicular form of papillary thyroid cancer (PTC) is associated with an increase in the incidence of the condition, with the follicular variant of PTC being the most common of all variants. The majority of individuals with the encapsulated follicular variant of papillary thyroid carcinoma (EFVPTC) are treated as though they have classic thyroid cancer, despite the availability of mounting evidence to contradict the aforementioned. According to numerous research, a certain type of noninvasive-EFVPTC (NI-EFVPTC) demonstrated poor histopathologic diagnostic reproducibility and has received aggressive treatment similar to that of a classical thyroid neoplasm. Therefore, to replace the term NI-EFVPC, a new nomenclature for these tumors, called "noninvasive follicular thyroid neoplasm with papillary-like nuclear characteristics" (NIFTP), was introduced in the year 2016. The present paper explores this recently introduced terminology, clinical, histologic, and molecular features, and diagnostic criteria.

## Introduction and background

Cancer that develops from thyroid parenchymal cells is called thyroid cancer. Despite the mortality rate remaining steady over the past few years, its incidence is progressively rising globally. Thyroid cancer has a wide range of clinical behaviors, from slow-growing, indolent, to extremely aggressive tumors with significant fatalities. In addition to evidence against overtreating thyroid malignancies with low risk, there are a number of state-of-the-art therapy options for advanced thyroid cancer [1]. Therefore, it is crucial to have a complete understanding of the different types of thyroid cancer and how to treat them in order to give the patient the right care.

The World Health Organization (WHO) 2017 Classification of Thyroid Tumors based on the histologic type and behavior of the tumor and papillary thyroid carcinoma (PTC) represent the commonest thyroid tumors [2]. PTC, a tumor termed for its papillary growth pattern, is entirely responsible for the growing incidence of this entity. However, the changes noted in the nuclear characteristics of the tumor cells are the prime diagnostic criteria. The most typical thyroid neoplasm, PTCs, has the best prognosis overall. This kind of epithelial malignancy exhibits follicular cell differentiation as well as a number of unique nuclear characteristics. The propensity of PTC to invade nearby structures, such as lymphatics, is one of its defining qualities. At the time of initial presentation, 10% of patients may have metastatic neoplasm. For the majority of patients, especially those under 45 years old, the prognosis is favorable [2].

There is a lot of interest in the epidemiology of PTC, as it accounts for 80-85% of all the thyroid tumors reported [2,3]. PTCs usually have a good prognosis with more than a 95% survival rate at 25 years. Patients are typically diagnosed with tumors during the third to fifth decades of life. When children and teenagers are affected by PTC, it often displays specific histopathologic characteristics that are uncommon in adults [3], in ratios of 2:1 to 4:1, with women being more frequently afflicted than men. The racial predilection is more towards the white race than the African race.

The incidence of PTC increased from 4.8 to 14.9 per 100,000 in a report based on the Surveillance, Epidemiology and End Results (SEER) database from 1975 to 2012 [4]. Several PTC subtypes have been identified, with classical PTC (cPTC) being the most prevalent (80%) and the follicular variant of PTC (FVPTC) being the second most prevalent subtype with 9% to 22.5% of PTC patients being affected by the same [5].

In the mid-1970s, the follicular form of PTC was widely recognized. This variant of PTC is made up of neoplastic follicles rather than papillae and the follicular cells display PTC-specific nuclear characteristics [6]. There are two primary subtypes that might exist: encapsulated and infiltrative/nonencapsulated. In the past two to three decades, the incidence of encapsulated FVPTC (EFVPTC) has likely increased by two to three folds, and it now accounts for 10% to 20% of thyroid cancer diagnoses in Europe and North America [7,8]. The encapsulated follicular variant of papillary thyroid carcinoma has a molecular profile more similar to follicular adenoma or follicular thyroid carcinoma than the infiltrative follicular variant of papillary thyroid carcinoma, which more closely resembles classical papillary thyroid carcinoma.

There has been a lot of debate over these discrepancies in encapsulated follicular form of papillary thyroid cancer, particularly centered on tumors that demonstrated no invasion. Therefore, this subgroup of FVPTC has lately been replaced by the term non-invasive follicular thyroid neoplasm with papillary-like nuclear characteristics (NIFTP) [9]. This modification was made to encourage more conservative treatment of these tumors and ensure that the patients are protected from the emotional strain of receiving a cancer diagnosis [9].

The present review paper will emphasize the disease presentation of NIFTP, diagnostic criteria, management, and future trends in the treatment of the same.

Non-invasive follicular thyroid neoplasm with papillary-like nuclear characteristics (NIFTP)

The non-invasive follicular thyroid neoplasm with papillary-like nuclear features (NIFTP) lacks well-formed papillae or psammoma bodies, as well as the typical findings of aggressive subtypes of PTC, and is a poorly differentiated tumor. It has a follicular growth pattern and nuclear features of PTC. NIFTP is considered to be a "borderline" or "pre-malignant" lesion [9].

Due to the tumor’s more indolent nature, the Endocrine Pathology Society proposed a new term in 2016 for this type of EFVPTC cases, "Noninvasive follicular thyroid neoplasm with papillary-like nuclear features (NIFTP)." They advocated for a conservative approach to treating this mostly benign illness, claiming that lobectomy alone, without adjuvant Radioactive Iodine (RAI), is sufficient to guarantee a patient long-term relief from recurrence [9].

All tumors that have previously been classified as noninvasive FVPTC would be considered NIFTP under this terminology and this has been officially approved by the WHO [9,10]. This approach to reclassification has offered a template for including other low-grade malignancies in addition to changing the categorization of a small but significant fraction of thyroid tumors. Furthermore, these lesions should actually be categorized as neoplasms because various molecular research shows that they are caused by clonal genetic changes [9].

## Review

Epidemiology

In their proposal to reclassify noninvasive EFVPTC as NIFTP, Nikiforov et al. projected that the new diagnosis would have an annual impact on more than 45,000 patients across the globe [9]. The projections produced a predicted rate of NIFTP of 18.6% among all PTC cases, depending on retrospective data from four institutions (three Italian and one American). It seems that the initial figures were inflated after more than three years since the new nomenclature was introduced [11,12]. According to a meta-analysis by Bychkov et al., nearly 9.1% of all PTC patients have NIFTP, which is significantly less common in the Asian population than it is in Western Europe and North America. Ethnic variation, variations in histological criteria interpretation, and clinical practice methods were all considered to be the prime reason for the reported disparity in NIFTP prevalence between Asians and non-Asian nations [13].

Clinical features of NIFTP

Similar to most thyroid neoplasms, NIFTP manifests clinically as a nodule during a routine physical examination or inadvertently during unrelated diagnostic imaging. If NIFTP becomes sufficiently large, it may also manifest by significant influence on nearby structures, resulting in dysphonia, globus feeling, or compromised airways [14].

Amidst a multinodular background, NIFTP manifests clinically as a single nodule or lesion. Two point seven percent (2.7%) to 14.7% of people with NIFTP had the multifocal type of disease. Fourteen point nine percent (14.9%) to 46.3% of NIFTP patients may also have a malignant lesion in the same or second lobe of the thyroid that frequently coexists with papillary thyroid microcarcinoma [15]. However, the final diagnosis is made only after the histopathological examination.

Radiological findings

NIFTP often appears as a well-defined and circumscribed, oval or round nodule upon ultrasound (US) investigation. Its echogenicity varies, ranging from notably hypoechoic to hypoechoic, isoechoic, and heterogeneous [16]. It is not surprising that there are such large variations in the observed echogenicity of NIFTPs since echogenicity is thought to have the lowest inter-observer concordance of all the features. It is uncommon to have calcifications within the lesions. NIFTPs typically reveal a mixed type of vascularization (peripheral and intranodular) during power Doppler ultrasound and are considered primarily to be hypervascular in nature [16,17].

Fine-needle aspiration biopsy

Fine-needle aspiration biopsy (FNAB) alone cannot be used to diagnose NIFTP and necessitates a postoperative histological diagnosis for confirmation. Nevertheless, some cytological characteristics found through FNAB may suggest NIFTP. The majority of the time, FNAB samples exhibit sparse colloids, numerous small clusters of follicular cells, microfollicles, and nuclear atypia [18]. Nevertheless, finding a papillary architecture would exclude the diagnosis of NIFTP.

NIFTP can be classified in any of the six categories of the Bethesda System for Reporting Thyroid Cytopathology (TBSRTC) in preoperative cytology; however, it is most frequently detected in the category that is referred to as “indeterminate” [18]. According to Bongiovanni et al.'s meta-analysis, the distribution of NIFTP in the TBSRTC categories is as follows: follicular neoplasm or suspicious for a follicular neoplasm (FN/SFN), 21%; nondiagnostic, 3%; benign, 10%; atypia of undetermined significance or follicular lesion of undetermined significance (AUS/FLUS), 30%; suspicious for malignancy (SM), 24%; and malignant, 8% [19].

The AUS/FLUS category should be subclassified according to the newly revised Bethesda classification. NIFTP is typically categorized as architectural atypia (AUS-A) or cytologic and architectural atypia when looking at the AUS/FLUS subcategories (AUS-C&A). It makes sense considering that microfollicles are a characteristic of NIFTP smears. Subcategories can assist in the prediction of the type of neoplasia. Furthermore, the subcategories of AUS-A and AUS-C&A can increase the concern about NIFTP and aid in choosing the right molecular testing [20].

Histopathological examination and diagnosis

Upon examination, NIFTP is considered a solid, well-defined, or encapsulated nodule. The thyroid tissue surrounding the lesion will be clearly differentiated from the surrounding thyroid tissue, despite the capsule's thinness and difficulty in being distinguished. In most cases, the cut surface is paler than the surrounding thyroid tissue's typical color, making necrosis and hemorrhage unlikely to be visible [9].

After submitting the entire tumor capsule for histology and carefully reviewing the nuclear characteristics and overall architecture, the histological diagnosis of NIFTP is determined by utilizing stringent inclusion and exclusion criteria [9].

In contrast to FVPTC, which has more irregular or lobulated borders and is primarily hypervascular, the ultrasound results of NIFTP are often those of an oval or round nodule that is well-circumscribed, hypervascular, and has a hypoechoic rim. Even though a preoperative diagnosis is not possible, it is crucial to be aware of thyroid nodules that may or may not correspond to NIFTP. If surgery is necessary for these nodules, lobectomy is advised unless total thyroidectomy is warranted for reasons other than the nodule's potential for cancer or worries about another nodule [16]. NIFTP can be diagnosed without the use of additional immunohistochemical stains besides hematoxylin and eosin; however, the presence of HBME-1, galectin-3, and CK19 in follicular-patterned tumors could establish a differential diagnosis that includes NIFTP and EFVPTC.

Only when both histopathological inclusion and exclusion criteria are met, the diagnosis of NIFTP is made (Table 1).

**Table 1:** Histological inclusion and exclusion criteria for the diagnosis of NIFTP

Inclusion criteria	Exclusion criteria
Growth pattern	Invasion
	Pattern of growth
	Papillary structures
Cytomorphonuclear features	Psammoma Bodies
	Tumor Necrosis
	Increased Mitosis
	Multifocality and size

Histopathologic criteria for inclusion

The Growth Pattern of NIFTP

NIFTP includes tumors that are completely encased with smooth muscle-walled vessels, partially encapsulated, and unencapsulated, but clearly distinct from surrounding parenchyma. Cystic tumors that exhibit both macro- and microfollicular architecture are possible. While the microscopic examination will reveal follicles with dense colloids in their centers, the majority of the tumor has a unique light grey-blue cast because of the PTC-like nuclear clearing that is typical of these tumors. But while some tumors have a small capsule, others have a thick fibrous one. In NIFTP, the intercalation of microfollicular foci more attenuated macrofollicular regions is prevalent [21,22].

Cytomorphonuclear Features

Importantly, to be diagnosed as NIFTP, these tumors must still exhibit nuclear characteristics of PTC, just as they did prior to the nomenclature amendment. Contrary to classical PTC, NIFTP nuclear characteristics are normally more subtly distributed and exhibit the so-called sprinkling sign (features ranging from diffuse to patchy and multifocal). Furthermore, they are frequently exacerbated toward the periphery of the tumor that is close to the capsule. Different degrees of intratumoral fibrosis may be present in NIFTP, and the colloid may be dense, hypereosinophilic, or watery [23].

In accordance with a generally accepted grading system, "papillary-like nuclear characteristics" must be present in order to qualify for the diagnosis. Include one point each for the presence of nuclear enlargement, overlap, crowding, elongation, nuclear membrane abnormalities, grooves, pseudo-inclusions, or glassy nuclei/marginated clear chromatin [23].

Histopathologic criteria for exclusion

Invasion

A diagnosis of NIFTP cannot be made if an invasion extends beyond a tumor's perimeter. This necessitates total tumor capsule penetration in encapsulated tumors, much like what would be necessary to identify invasion in follicular thyroid cancer. The presence of lymphatic and/or vascular invasion also rules out this diagnosis. This is indicated by tumor cells that are typically accompanied by a thrombus or exhibit wall attachment and are located within an endothelium-lined area in the capsule or arteries outside the tumor [24].

NIFTP nomenclature is not advised in the uncommon circumstance where the tumor capsule is surgically removed, leaving the tumor at the margin but not necessarily extrathyroidal in extent. The tumor-to-normal tissue interface should be studied because a diagnosis of NIFTP necessitates a lack of invasion to make the final diagnosis [23].

The Pattern of Growth

The architecture of the tumor is predominately follicular. Rarely, a small portion of solid, trabecular, or insular architecture may be visible in tumors. These additional growth patterns, however, are limited to no more than 30% of the total tumor volume. NIFTP would not be the diagnosis if there is a presence of other particular morphologies such as tall or columnar cell features, or cribriform architecture. Furthermore, NIFTP should not be used as the diagnosis if it is difficult to interpret the pattern of growth [25,26].

Papillary Structures

Initially, the NIFTP classification was appropriate for single, isolated papillae that made up less than 1% of the tumor volume. However, this classification is indeed arbitrary. It is currently advised that the NIFTP spectrum should contain NO well-formed papillae. A papillary structure is, by definition, a fibrovascular core surrounded by malignant follicular cells. Histological confirmation of fibrovascular cores is necessary, showing fine endothelial cells encircling erythrocytes. Sanderson's polsters, which are assemblages of tiny follicles that protrude into the lumen of follicles but lack true fibrovascular cores, do not meet the criteria for being considered actual papillae [11].

Psammoma Bodies

When referring to papillary thyroid cancer, papillae that have undergone concentric calcium lamination are represented by psammoma bodies, which are mummified and dead. As a result, the presence of psammoma bodies would indicate the existence of papillae, which should by definition be incredibly uncommon in NIFTP. Additionally, finding a psammoma body in the lymphatics or lymph nodes' fibrous septae is indicative of lymphatic invasion. Psammoma bodies in lymphatics should be interpreted as evidence of invasion in the absence of another reasonable explanation, excluding the tumor from the NIFTP category [27].

Tumor Necrosis

The use of NIFTP would be prohibited in any regions with "real" tumor necrosis, which is defined as comedonecrosis or confluent necrosis. These high-grade characteristics will exclude the diagnosis of NIFTP. The presence of the neoplastic cells' ill-defined outlines typically indicates actual necrosis [28].

Mitoses

Similar to necrosis, finding more mitoses (having more than three mitoses per 10 high-power fields) is typically regarded as a high-grade feature. This would prevent the use of NIFTP when combined with solid, trabecular, insular growth, and necrosis [23].

Multifocality and Size

It is significant to remember that any thyroid gland tumor may exhibit tumor multifocality. As a result, the diagnosis of another topographically distinct tumor in the gland should not be excluded by the presence of NIFTP. NIFTP is not included in the staging process, however, as only malignant tumors would be taken into account [23].

Molecular characteristics of NIFTP

The thyroid TCGA findings showed that PTC can be broadly categorized into subgroups that resemble BRAF V600E and RAS. NIFTP, which has a genetic profile similar to that of RAS, has activating RAS mutations in 30-54% of patients, including NRAS, HRAS, and, infrequently, KRAS mutations. Additionally, a small portion of NIFTP has gene rearrangements in PPARG, a molecular change first connected to FTC, and THADA rearrangements. BRAF variants other than V600E, namely, the BRAF K601E mutation, have also been detected in NIFTP [29].

Diagnostic criteria for NIFTP

Nikiforov YE et al., in 2016, conducted an international, interdisciplinary, retrospective study comprising patients with thyroid nodules diagnosed as EFVPTC. The objective of the study was to evaluate the clinical outcome, ascertain a diagnostic criterion, and institute a terminology that accurately reflects the clinical and histopathologic features of EFVPTC [9]. One hundred nine (109) patients with noninvasive EFVPTC were followed for a period of 10 to 26 years, and 101 participants with invasive EFVPTC and observed for a period of one to 18 years were included in the aforementioned study. The frequency of adverse events, such as mortality rate related to the disease, local or distant metastases, and recurrence of the neoplasm, among patients with noninvasive and invasive EFVPTC was identified using a set of repeatable histopathologic criteria. These criteria served as the primary outcome measures of the study [9].

Twenty-four thyroid pathologists collaborated to create consensus diagnostic standards for EFVPTC. The term "noninvasive follicular thyroid neoplasm with papillary-like nuclear characteristics" (NIFTP) was chosen based on the outcomes data for noninvasive EFVPTC [9]. The suggested diagnostic criteria for noninvasive EFVPTC are shown in Table 2. With NIFPT no longer regarded as "cancer," there is a decrease in the risk of "malignancy" in thyroid nodules with the characteristics described in Table 3, which are commonly identified in lesions correlating to NIFTP [30,31].

**Table 2:** The proposed new nomenclature for the diagnosis of EFVPTC EFVPTC: encapsulated follicular variant of papillary thyroid carcinoma Adapted from [9]

Major Features	Minor Features
Encapsulation or clear demarcation, Follicular growth pattern, Nuclear features of papillary thyroid carcinoma (PTC)^a^: Enlargement, crowding/overlapping, Elongation, Irregular contours, Grooves, Pseudoinclusions^b^, Chromatin clearing^c^	Dark colloid, Irregularly shaped follicles, Intratumoral fibrosis, “Sprinkling” signs, Follicles cleft from stroma, Multinucleated giant cells within follicles
Features Not Seen/Exclusion Criteria: no “True” papillae, Psammoma bodies, Infiltrative border, Tumor necrosis, High mitotic activity^f^, Cell/morphologic characteristics of other variants of PTC^g^	
^a^ Most pathologists do not mandate a particular percentage of the tumor nodule to exhibit these signs in a tumor with a multifocal presence of PTC. ^b^ A significant and valuable diagnostic feature for all PTC types, however, infrequent in EFVPTC. ^c^ "Glassy" or "Orphan Annie" nuclei are defined as those in which the usual chromatin distribution has been erased and the chromatin shows margination towards the membrane. ^d^ As described and illustrated by Vanzati et al [31] ^e^,according to definitions, true papillae are complex, arborizing papillae with fibrovascular cores, bordered by cells that exhibit nuclear characteristics of PTC, and disassociated from a region suitable for FNAB. ^f^ At least 3 per 10 high-power fields (×40). ^g^ Such as tall cell features, cribriform-morular variant, solid variant, etc.	

**Table 3:** Data that suggest a thyroid nodule matching NIFTP reasonably could exist Adapted from [31] NIFTP: non-invasive follicular thyroid neoplasm with papillary-like nuclear characteristics

Criteria	Findings
Clinical examination	No clinically obvious metastasis of the tumor originated from the thyroid^a^
Ultrasonography	Absence of lymph node metastases^a^, Nodule without the following findings: Extrathyroidal extension, Microcalcification taller-than-wide shape, Spiculate/microlobulate/ill-defined margin, Increased likelihood of malignancy
Fine-needle aspiration	Category III, IV or V cytology of the Bethesda system^b^, Nodule without mutations or with RAS or other RAS-like mutations (e.g., PAX8/PPARG rearrangement)^c^
^a^The presence either suggests an associated thyroid tumor or rules out NIFTP.; ^b ^Follicular pattern, no papillae, no psammomatous calcifications, no florid nuclear features of papillary thyroid carcinoma, no necrosis or mitoses; ^c^ When a nodule has BRAFV600E or other BRAFV600E-like mutations (e.g., RET/PTC fusions), high-risk mutations, such as TERT promoter, p53, NIFTP is essentially ruled out.	

The essential histopathologic characteristics of this lesion are lack of invasion, follicular growth pattern, and nuclear hallmarks of PTC, and these features are reflected in the newly proposed terminology, NIFTP. A small number of samples were used for the molecular analysis in this investigation, and the results supported earlier findings. It was found that the majority of these lesions are caused by genetic mutation and neoplastic proliferation of the cells. These tumors are not anticipated to exhibit genetic abnormalities linked to classic PTC, such as BRAFV600E mutations, when defined with rigorous histopathologic criteria. Alternatively, the cells exhibited an increased incidence of RAS and other mutations that have previously been connected to a follicular variant of thyroid tumors [9].

Additionally, the propensity for invasion and the ability of the tumor to reiterate the follicular adenoma to follicular thyroid carcinoma progression sequence suggests that NIFTP exemplifies the benign counterpart or predecessor of the invasive EFVPTC.

Ever since the introduction of the 2016 nomenclature, several micrometastases of local lymph node cases and tumors that appear to match the recommended diagnostic criteria for NIFTP have been documented. Therefore, to overcome this limitation, Nikiforov YE et al. in 2018 advocated replacing the criterion of "less than 1% papillae" with the criterion of "no well-formed papillae" to prevent misdiagnosing these tumors as NIFTP. Table 4 details the modification to the diagnostic criteria for nuclear features akin to NIFTP [32].

**Table 4:** Revised diagnostic criteria for NIFTP proposed by Nikiforov YE in 2018 Adapted from [32] NIFTP: non-invasive follicular thyroid neoplasm with papillary-like nuclear characteristics

Primary	Secondary^e^
Encapsulation or clear demarcation^a^, Follicular growth pattern with: No well-formed papillae, No psammoma bodies, <30% solid/trabecular/insular growth pattern, Nuclear score 2-3^b^, Absence of vascular or capsular invasion^c^, Absence of tumor necrosis or high mitotic activity^d^	Absence of BRAF V600E mutation noticed during molecular assays or immunohistochemistry, No BRAF V600E-like mutations or other high-risk mutations (TERT, TP53)
^a^ Thick, thin, partial, or well-circumscribed capsule having a distinct delineation from contiguous thyroid parenchyma. ^b^ Usually, a nuclear score is 2 (amply expressed nuclear features of PTC). To rule out the papillae presence, the complete tumor sample should be investigated in PTC tumors with nuclear score 3 (florid nuclear characteristics). Immunohistochemistry for BRAF V600E or molecular testing for BRAF V600E and other mutations are recommended but not necessary for lesions with nuclear score 3. ^c^ Entire tumor capsule interface must be examined under a microscope. ^d^ When there are 3 or more mitoses for every 10 high-power fields (×400), high mitotic activity is significant. ^e^ Secondary criteria are beneficial but not necessary for the diagnosis of NIFTP.	

Additionally, the new nomenclature highlighted that in tumors with pronounced nuclear signs of PTC, evaluation of the complete tumor should be done in addition to the rigorous need for inspection of the entire tumor capsule in order to rule out the presence of papillary structures. This suggestion is based on the observation that NIFTP more typically exhibits nuclear features of PTC that are moderately developed, and that their overt presence should prompt suspicion of classic PTC [32].

Management of NIFTP

The American Thyroid Association’s Thyroid Nodules and Differentiated Thyroid Cancer (ATATNDTC) guidelines state that patients with NIFTP tumors are subject to the same recommendations as patients with low-risk differentiated thyroid carcinoma. The recommendation suggested that lobectomy is sufficient, remnant ablation is not advised, and it is mandatory that thyrotropin target levels should be maintained between 0.5 and 2 mIU/L [33]. A total thyroidectomy is still a viable option for some NIFTP patients, despite the fact that the precise diagnosis of the condition can only be determined through postoperative histological investigation. Physical examination, ultrasound findings, and molecular features should all be taken into consideration when deciding on the type and extent of surgical treatment [34].

Bilateral disease extension and multifocal type of the disease are the two key factors of NIFTP neoplasms that should be taken into account when deciding how much surgery is necessary [35,36]. In particular, when considering a lobectomy, the individual should be made aware of the possibility of a second surgery. Based on the aforementioned information, a surgeon decides whether to opt for lobectomy or complete thyroidectomy.

As of now, there are no recommendations for monitoring patients diagnosed with NIFTP. According to the ATATNDTC guidelines, occasional monitoring of the patient with the estimation of serum Tg and neck ultrasound can be considered the standard follow-up protocol. Tg levels <2 ng/mL and <10 ng/mL in patients who underwent surgical resection and lobectomy, respectively; the absence of anti-Tg antibodies and normal findings during neck ultrasonography are regarded as the prerequisites to rule out future retesting for the recurrence of the lesion [37].

Future perspectives and recommendations

The term NIFTP denotes a slow-growing lesion and the course of the disease avoids overtreatment of the lesion. This raises concerns about the number of NIFTPs that may have been overlooked by US/FNAB workups, which are further complicated by the non-resection of the lesion, which would exclude histological confirmation. Therefore, it is conceivable that the true incidence of NIFTP may continue to be underreported. Future studies should evaluate how to enhance US/FNAC workups to find NIFTP [38].

Also, the therapeutic options for NIFTP are limited, with no noninvasive and nonsurgical management of the tumor. Among the surgical resection procedures, lobectomy and total thyroidectomy are the treatment choices. Even if lobectomy is sufficient for the treatment of NIFTP, some patients still have the choice of a total thyroidectomy, especially if they refuse to accept the requirement for monitoring the residual thyroid lobe and the possibility of signs and symptoms necessitating a second surgery. Therefore, the prime focus of future research must be on pioneering contemporary and advanced treatment options for the management of NIFTP tumors [38].

Though many retrospective studies have shown a potential minimal risk of nodal and distant metastatic disease in tumors categorized as NIFTP, large-scale population-based studies with long-term clinical follow-ups are required to fully describe the behavior of these tumors [39].

## Conclusions

The inclusion of NIFTP in the most recent WHO endocrine tumor classification system demonstrates the widespread acceptance of this lesion. The recent classification and change in the nomenclature of thyroid carcinomas provide clarity on diagnosing and categorizing different types of thyroid tumors based on their invasiveness. More research will be helpful in the future to fully characterize the molecular findings of this lesion and establish evidence-based literature on the long-term follow-up of these tumors. Even though the diagnosis is postoperative, a thorough preoperative evaluation may raise NIFTP suspicion and point to a more conservative course of treatment.
